# Visual Hallucinations as Incidental Negative Effects of Virtual Reality on Parkinson's Disease Patients: A Link with Neurodegeneration?

**DOI:** 10.1155/2015/194629

**Published:** 2015-05-10

**Authors:** Giovanni Albani, Elisa Pedroli, Pietro Cipresso, Daniel Bulla, Veronica Cimolin, Astrid Thomas, Alessandro Mauro, Giuseppe Riva

**Affiliations:** ^1^Division of Neurology and Neurorehabilitation, Ospedale San Giuseppe, IRCCS Istituto Auxologico Italiano, Via Cadorna 90, Piancavallo, 28824 Verbania, Italy; ^2^Applied Technology for Neuro-Psychology Laboratory, IRCCS Istituto Auxologico Italiano, Via Magnasco 2, 20149 Milano, Italy; ^3^Department of Electronics, Information and Bioengineering, Politecnico di Milano, Piazza Leonardo da Vinci 32, 20133 Milano, Italy; ^4^Department of Neurology, Neuroimaging and Medical Sciences, Università degli Studi “G. d'Annunzio” di Chieti-Pescara, Via dei Vestini 31, 66100 Chieti, Italy; ^5^Department of Neuroscience “Rita Levi Montalcini”, Università di Torino, Via Cherasco 15, 10126 Torino, Italy; ^6^Department of Psychology, Università Cattolica di Milano, Largo Gemelli 1, 20123 Milano, Italy

## Abstract

We followed up a series of 23 Parkinson's disease (PD) patients who had performed an immersive virtual reality (VR) protocol eight years before. On that occasion, six patients incidentally described visual hallucinations (VH) with occurrences of images not included in the virtual environment. Curiously, in the following years, only these patients reported the appearance of VH later in their clinical history, while the rest of the group did not. Even considering the limited sample size, we may argue that VR immersive systems can induce unpleasant effects in PD patients who are predisposed to a cognitive impairment.

## 1. Introduction

Nowadays, there is an increasing emphasis on the application of technology in the assessment and rehabilitation of neurological diseases and one of these technologies is Virtual Reality (VR). While the literature is limited, there are research programs studying this application specifically in Parkinson's disease (PD). As such, understanding the usefulness, the limitations, and the considerations in the application of the technology is important to both scientific and clinical communities.

In the past few years, strong scientific production has sustained the role played by external cues, such as visual ones, in the multisensory rehabilitative approach to PD [1].

VR technologies combined with treadmill training have been successfully used to ameliorate gait in PD [2–4] by means of an attention-trigger strategy even if, in some cases, a beneficial effect has been described as limited to PD subjects at advanced stages of the disease [5, 6].

An occasional negative impact of immersive VR systems on PD patients has been preliminarily reported also for the postural control [7].

We previously [8, 9] reported the incidental experience of visual hallucinations (VH) in six of 23 PD patients during immersion in a VR environment in which their virtual actions were tested in comparison with controls.

These observations generate the need to find a clinical reason that may explain the unpleasant effects of VR immersion and help us better understand where the positive effects of VR technology end and the negative ones start.

A relationship between VH and cognitive impairment in PD has been tentatively established: PD patients with VH show a prolonged choice reaction and a stimulus discrimination deficit [10], as well as an increased risk of dementia [11].

Aim of the present study was to study retrospectively after eight years all the 23 cases who participated in the first VR study [9], in order to verify if that VH reported during the virtual session may have some correlation with the actual cognitive status.

## 2. Methods

In the 2005 study [8, 9], the aim was to evaluate the behaviour of 23 PD patients and 15 healthy controls in VR environments reproducing daily living situations (i.e., supermarket, gym, and kitchen) both after one-hour L-dopa (LD) intake and 12 hours after withdrawal of the LD.

PD patients performed their virtual actions worse than the controls in terms of time of execution, exploration, pointing, and precision in avoiding obstacles. None of the controls complained of any disturbances, such as minor transient effects (nausea or vertigo) or visual abnormalities.

Although none of the participants reported ever having experienced VH, during the off-state virtual session, six PD patients incidentally reported an occurrence of images not included in the virtual environment (Table 1).

The “object” of VH was the same for all patients. According to their descriptions, it seems that in all cases the patient preserved the insight that the hallucinated image was inappropriate at that point of virtual navigation, but they did not appear surprised or worried about this visual experience.

We reviewed the clinical history of all six patients who experienced VH: none had previously reported anything similar to illusions or sensations of presence or passage.

In 2013, we tried to follow up all 23 PD patients of the previous study. According to an agreement between the Local Association of Patients and the Division of Neurology and Neurorehabilitation of the Istituto Auxologico Italiano Piancavallo (Verbania), Italy, all patients may be visited at least once per year. The visits included a clinical update via a neurological exam (UPDRS motor part) [12] and a neuropsychological battery, including Mini Mental State Examination [13], Frontal Assessment Battery [14], and the Neuropsychiatry Inventory Test [15].

Patients were retrospectively divided into two groups according to the results of the first study: with VH (VRVH, 6 patients) and without VH (WVRVH, 17 patients).

**Figure 1:**
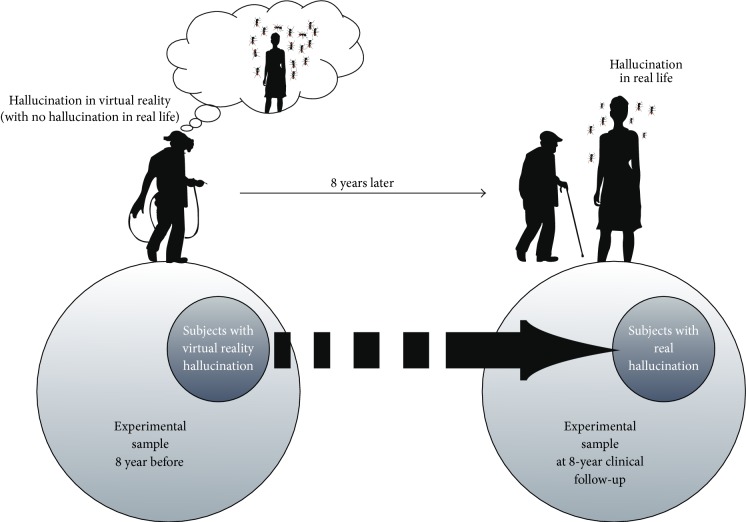
Experimental design.

In the VRVH group, one patient had died and one had undergone deep brain stimulation surgery (but was still included in the follow-up); in the second group (WVRVH), four patients had died and two patients could not be found. Thus, 5 of the 6 patients in the VRVH group and 11 of the 17 patients in the WVRVH group completed the follow-up (Figure 1).

## 3. Results

While none of the patients in the WVRVH group presented any hallucination in their clinical history, all six patients of the VRVH group reported the appearance of VH during the course of their disease. Curiously, the dead patient in the first group developed VH during their clinical course, while the four dead patients of the second group did not.

**Figure 2:**
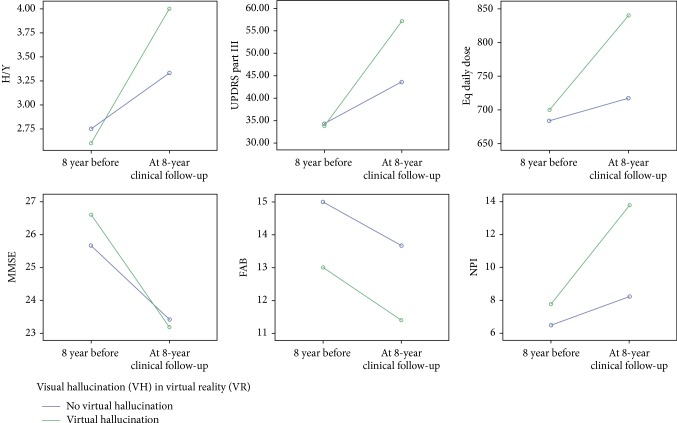
All variables show that all the patients eight years later worsened, but even more so for the patients who experienced virtual hallucinations after eight years.

Demographics and clinical characteristics of the groups at baseline and eight years later at the follow-up are shown in Table 2 and Figure 2.

In Table 3, the classification table by logistic regression shows no prediction of VH starting from neuropsychological tests, while in Table 4 all the variables out of the logistic regression equation are reported. From Table 3 it can be seen that the neuropsychological tests (also reported in Table 4) were not able to predict (based on the logistic regression) VH* a priori*, highlighting that only VR was able to make a prediction in this sense. In fact, according to neuropsychological tests, the logistic regression forecasted that no patients would report VH; however, during VR six patients incidentally described VH confirmed eight years later. This result makes how VR highlighted a phenomenon that cannot be predicted with standard neuropsychological tests evident.

## 4. Discussion

Even with the limited sample size in this study, we reported a correlation between complaining of VH during a VR immersion and further appearance of VH in the clinical picture.

This finding leads us to two relevant observations concerning VR technology: (1) it can potentially induce unwanted effects in PD patients; and (2) there are questions about its usefulness in predicting further development of VH in PD.

### 4.1. Visual Hallucinations as Unwanted Effects of Virtual Reality

To our knowledge, in the literature there are no reports of unwanted effects concerning VR sessions for PD patients; our findings seem to be in contrast with the increasing claims of usefulness of VR in the rehabilitation approach of these patients [2–4, 16].

One explanation is that most VR methodologies used in rehabilitation programs for PD are not immersive or projected-based systems that strongly reduce the sense of presence, which may be considered a trigger for unwanted effects, especially those regarding the visual system.

VR experiences are pooled by a common step-simulation by the interaction [17]. One of the most critical features that differentiates immersive VR from nonimmersive VR is the sense of reality (better known as “sense of presence” among experts in cybertherapy), which means that the proband feels the virtual experience as real.

This feeling of presence, which in adult subjects is modulated by the prefrontal cortex-visual system loop [18], is strictly influenced by the sophistication of the used software.

### 4.2. Why Visual Hallucinations?

Various pathogenic models have been considered to explain VH in PD, such as the hypersensitivity of mesolimbic receptors due to dopaminergic denervation during a dopaminergic treatment [19], the deregulation of external perception and internal image production according to the dream imagery intrusion model [20], a deficit in reality monitoring [21], or a processing disruption across attentional networks [22].

VH are one of the most typical symptoms among the behavioural disturbances observed in PD, affecting about one-quarter of patients. Several descriptions reported the presence of formed images as inanimate objects or indefinite/not fully formed images, sensation of presence (persons-guardian angel), or simply illusions consisting of the misinterpretation of images, with the overlap of humanoid or animal tracts on animated objects [23].

Curiously, VH can also include the feeling of an abnormal “presence” (a vague and erroneous perception that another person or threat is present) or “passage” hallucinations (transient undefined hallucinations that pass through the periphery of the visual field) [24].

The presence of VH during VR immersion is an interesting and unexpected finding, as few side effects following immersion in VR have been previously reported in literature. Research conducted on a group of healthy individuals evidenced transient reduced binocular vision after wearing a head-mounted display for just ten minutes [25], which was probably due to the generation of three-dimensional visual space from two-dimensional images. Nausea has been reported during 20-minute immersion periods and ten-minute postimmersion periods, which has been attributed to the incongruity between visual and vestibular motion cues [26].

VH in VR navigation show similarities with VH usually evoked in the clinical history of PD patients, such as preserved awareness, only a moderate impact of stress, and the incidence of images resembling animals (three of six patients in the present study).

The VH shown by our patients always occurred only during “off-medication” periods.

Other authors [27] reported an impaired adaptation to visuomotor perturbations in VR environments during off-state, suggesting a hypofunctional state of the dopaminergic retinal system and reflecting the same “on-off” dependence alterations of visual abnormalities observed during recording by contrast sensitivity, electroretinograms, and visual evoked potentials in PD patients with motor fluctuations [16]. Furthermore, it was also shown that vision fluctuates in parallel with motor fluctuations [28]. Some authors also reported that VH tend to occur during times of low ambient stimulation, most typically in the evening or when the patient is alone in a quiet environment [29]. Finally, visual attention impairment has been described in patients with VH with acquired eye diseases [30].

We can argue that a reduced dopaminergic state is correlated with an altered sense of presence, such as that observed in children whose immature prefrontal brain structure would lead them to an increased susceptibility for the experience of presence [22]. Thus, as also reported by neurofunctional studies [31], a dysfunction of the frontal areas associated with the control of visual attention could predispose PD patients to VH through an abnormal processing of relevant and irrelevant visual stimuli.

### 4.3. Visual Hallucinations as Prediction of Cognitive Impairment

Various prospective studies [11, 32, 33] have considered VH a risk factor for dementia. When examined longitudinally, VH affect more patients than is commonly assumed in cross-sectional prevalence studies. Neuropsychological and FDG-PET studies have demonstrated that visuoperceptual impairment and early involvement of the posterior neocortex may be risk factors for rapid symptomatic progression and dementia in PD [34].

In the present study, the comparison between the two groups shows that the L-dopa eq/die of the WVHVR patients is substantially lower at the baseline than in the follow-up, so that the Hoehn/Yahar stage and UPDRS part III score. These data would support the assumption that visual hallucinations, cognitive impairment, and motor scores seem to run in the same direction [10, 11].

However, our findings can only raise and not solve the question of whether VR technology can potentially be considered a useful tool to predict who could further develop VH.

Indeed, our study is limited by the restricted number of cases and by the fact that a repetition of the VR session was not included in the clinical follow-up. Moreover, as the VH were an unexpected side effect at that time, visual performances (such as color discrimination and contrast perception) of all patients have not been investigated.

## 5. Conclusions

In consideration of the growing use of VR in PD treatment, the first intent of this paper is to alert the scientific community about the possibility of unwanted effects when immersive systems are used.

Moreover, we describe a link between past and further occurrence of VH in VR and clinical history, respectively, but only further controlled studies with larger numbers of cases will lead to the answers.

## Figures and Tables

**Table 1:** Location and characteristics of the hallucinations.

Patient number	Location and characteristics of the hallucinations
1	In gymnasium: unidentified animals on the wall
2	In gymnasium: a nest of bees, with the bees flying around
3	In gymnasium: children sitting at desk
4	In kitchen: a petrol pump
5	In supermarket: the woman is mistaken for a policeman
6	In supermarket: unidentified animals on the wall and on the floor

**Table 2:** Demographics and clinical characteristics of groups at baseline and eight years later at follow-up.

	VHVR (6)	WVHVR (17)	*P *
Age baseline	67 ± 7.5	65 ± 8.3	NS

Sex (m)	1	8	NS

Education	8.5 ± 3.8	9.5 ± 5.4	NS

MMSE	Baseline 26.5 ± 1.7 Follow-up 23.2 ± 3.3	Baseline 25.6 ± 2.9 Follow-up 22.3 ± 3.7	NS NS

Hoehn/Yahr stage	Baseline 2.8 ± 1.5 Follow-up 4 ± 0	Baseline 2.8 ± 1.1 Follow-up 3.1 ± 0.7	NS *P* < 0.005

UPDRS part III	Baseline 34.1 ± 6.3 Follow-up 51.2 ± 7	Baseline 34.9 ± 14.4 Follow-up 40.3 ± 11	NS *P* < 0.007

L-dopa eq/die (mg/day)	Baseline 683.3 Follow-up 695	Baseline 788 Follow-up 633	NS NS

DA therapy (%)	Baseline 100 Follow-up 100	Baseline 30 Follow-up 70	

VH (%)	Baseline 0 Follow-up 100	Baseline 0 Follow-up 0	

NPI	Baseline 9.8 ± 8.20 Follow-up 13.8 ± 7.8	Baseline 6.8 ± 5.8 Follow-up 8.3 ± 7.1	NS NS

FAB	Baseline 12.8 ± 3 Follow-up 11.4 ± 2.3	Baseline 14.6 ± 2.7 Follow-up 13.4 ± 2.6	*P* < 0.04 *P* < 0.07

All values represent mean (SD, when not otherwise stated).

MMSE = Mini Mental State Examination; NPI = Neuropsychiatry Inventory; FAB = Frontal Assessment Battery; UPDRS part III = Unified Parkinson's Disease Rating Scale-subscale III; VH = visual hallucination in the real life.

**Table 3:** Classification table by logistic regression showing no prediction of visual hallucination starting from neuropsychological tests.

Observed	Predicted		
	No virtual hallucination	Virtual hallucination	Percentage correct
No virtual hallucination	17	0	100.0
Virtual hallucination	6	0	0.0

**Table 4:** Table showing that all the variables are not in the logistic regression equation.

		Score	df.	Sig.
Variables	Education	.184	1	.668
	H/Y stage	.335	1	.563
	UPDRS part III	.017	1	.895
	Eq daily dose	.007	1	.935
	MMSE	.461	1	.497
	FAB	2.837	1	.092
	NPI	1.030	1	.310

Overall statistics		**5.244**	**7**	**.630**
